# Tie2 activation protects against prothrombotic endothelial dysfunction in COVID-19

**DOI:** 10.1172/jci.insight.151527

**Published:** 2021-10-22

**Authors:** Alec A. Schmaier, Gabriel M. Pajares Hurtado, Zachary J. Manickas-Hill, Kelsey D. Sack, Siyu M. Chen, Victoria Bhambhani, Juweria Quadir, Anjali K. Nath, Ai-ris Y. Collier, Debby Ngo, Dan H. Barouch, Nathan I. Shapiro, Robert E. Gerszten, Xu G. Yu, MGH COVID-19 Collection and Processing Team, Kevin G. Peters, Robert Flaumenhaft, Samir M. Parikh

**Affiliations:** 1 Division of Cardiovascular Medicine, Beth Israel Deaconess Medical Center and Harvard Medical School, Boston, Massachusetts, USA.; 2 Department of Medicine, Beth Israel Deaconess Medical Center, Boston, Massachusetts, USA.; 3 Ragon Institute of MGH, MIT and Harvard, Cambridge, Massachusetts, USA.; 4 Division of Pulmonary, Critical Care and Sleep Medicine, Beth Israel Deaconess Medical Center and Harvard Medical School, Boston, Massachusetts, USA.; 5 Massachusetts General Hospital and Harvard Medical School, Boston, Massachusetts, USA.; 6 Cardiovascular Research Center, Beth Israel Deaconess Medical Center, Boston, Massachusetts, USA.; 7 Department of Obstetrics and Gynecology,; 8 Center for Virology and Vaccine Research, and; 9 Department of Emergency Medicine, Beth Israel Deaconess Medical Center and Harvard Medical School, Boston, Massachusetts, USA.; 10 Infectious Diseases Division, Brigham and Women’s Hospital and Harvard Medical School, Massachusetts, Boston USA.; 11 The MGH COVID-19 Collection and Processing Team is detailed in Supplemental Acknowledgments.; 12 Aerpio Pharmaceuticals, Inc., Cincinnati, Ohio, USA.; 13 Division of Hemostasis and Thrombosis and; 14 Division of Nephrology, Beth Israel Deaconess Medical Center and Harvard Medical School, Boston, Massachusetts, USA.; 15 Division of Nephrology, University of Texas Southwestern, Dallas, Texas, USA.

**Keywords:** COVID-19, Vascular Biology, Coagulation

## Abstract

Endothelial dysfunction accompanies the microvascular thrombosis commonly observed in severe COVID-19. Constitutively, the endothelial surface is anticoagulant, a property maintained at least in part via signaling through the Tie2 receptor. During inflammation, the Tie2 antagonist angiopoietin-2 (Angpt-2) is released from endothelial cells and inhibits Tie2, promoting a prothrombotic phenotypic shift. We sought to assess whether severe COVID-19 is associated with procoagulant endothelial dysfunction and alterations in the Tie2/angiopoietin axis. Primary HUVECs treated with plasma from patients with severe COVID-19 upregulated the expression of thromboinflammatory genes, inhibited the expression of antithrombotic genes, and promoted coagulation on the endothelial surface. Pharmacologic activation of Tie2 with the small molecule AKB-9778 reversed the prothrombotic state induced by COVID-19 plasma in primary endothelial cells. Lung autopsies from patients with COVID-19 demonstrated a prothrombotic endothelial signature. Assessment of circulating endothelial markers in a cohort of 98 patients with mild, moderate, or severe COVID-19 revealed endothelial dysfunction indicative of a prothrombotic state. Angpt-2 concentrations rose with increasing disease severity, and the highest levels were associated with worse survival. These data highlight the disruption of Tie2/angiopoietin signaling and procoagulant changes in endothelial cells in severe COVID-19. Our findings provide rationale for current trials of Tie2-activating therapy with AKB-9778 in COVID-19.

## Graphical abstract

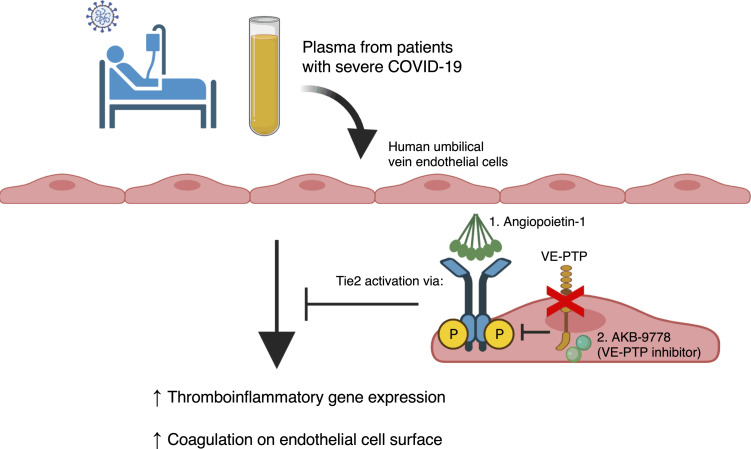

## Introduction

Severe acute respiratory syndrome coronavirus 2 (SARS-CoV-2), the virus responsible for coronavirus disease 2019 (COVID-19), can result in critical illness characterized by severe pulmonary disease (1), in addition to several extrapulmonary manifestations (2), and carries a significant morality rate. Critical COVID-19 illness is characterized by a prothrombotic coagulopathy, and higher D-dimer concentrations and activation of coagulation are associated with worse outcomes (3–5). Fibrin deposition in the lung vasculature is a commonly identified histopathologic finding, and microvascular thrombosis may be a key driver of COVID-19 pathophysiology (6, 7). Multiple lines of evidence, including measurement of circulating proteins and metabolites and analysis of histologic specimens, have demonstrated severe vascular inflammation and endothelial injury (8). Therefore, a phenotypic switch of endothelial cells to a procoagulant state appears to be a critical disease mechanism. Endothelial dysfunction in COVID-19 may be mediated through circulating inflammatory cytokines (9, 10), autoantibodies (11, 12), and neutrophil extracellular traps (NETs) (13), as well as potentially via direct viral infection (14).

Procoagulant changes in endothelial cells can be characterized by loss of constitutive anticoagulant function and/or upregulation of thromboinflammatory mediators. Several studies have demonstrated that severe COVID-19 is associated with increased circulating levels of such endothelial markers, including procoagulant von Willebrand factor (vWF) (15), plasminogen activator inhibitor (PAI-1) (16), vascular cell adhesion markers (17–19), and increased circulating antithrombotic endothelial surface proteins tissue factor pathway inhibitor (TFPI) (20) and thrombomodulin (21), which are cleaved off the endothelial surface during inflammation (22).

We have previously demonstrated via unbiased proteomics that endothelial dysfunction is strongly implicated in the coagulopathy of critical illness (23). Specifically, dysregulation of the Tie2-angiopoietin pathway emerged as a central link between vascular inflammation and inappropriate coagulation (23). The receptor tyrosine kinase Tie2 is highly enriched in vascular endothelium, and its ligand angiopoietin 1 (Angpt-1) promotes vascular stability and quiescence by activating Tie2 signaling (24). Moreover, activation of the Tie2 pathway can prevent the heightened thrombosis that occurs during septic endothelial injury (23). Angiopoietin 2 (Angpt-2) is an Angpt-1 paralog that competitively inhibits Tie2. Angpt-2 expression is promoted by tissue hypoxia and other inflammatory pathways and is subsequently stored in endothelial Weibel-Palade bodies and released during endothelial activation (24). Angpt-2 levels are even further elevated in the plasma of patients with sepsis with disseminated intravascular coagulation compared with those with sepsis alone, and high Angpt-2 levels potentiate endothelial dysfunction of critical illness (23, 24). Loss of constitutive Tie2 signaling is mediated through Angpt-2 antagonism or by Tie2 cleavage from the endothelial surface (25, 26). Inhibition of Tie2 results in loss of barrier function and anti-inflammatory transcriptional machinery, most notably characterized by upregulation of Angpt-2 itself, leading to a positive feedback loop and further Tie2 suppression. Tie2 kinase activity is also tonically inhibited by vascular endothelial protein tyrosine phosphatase (VE-PTP). Similar to Angpt-2, VE-PTP expression is increased under conditions of endothelial stress such as hypoxia, creating an Angpt-1–resistant state (27, 28). A first-in-class VE-PTP inhibitor small molecule, AKB-9778 (Razuprotafib) restores cytoprotective Tie2 signaling to protect endothelial cell function in the presence of inflammatory stimuli (27, 29, 30).

Since Angpt-2 is closely linked with the coagulopathy of sepsis (23), we hypothesized that Angpt-2 may also be associated with the host endothelial response in COVID-19 and, more importantly, represent a therapeutic target. We used a cell culture model to perturb the Tie2-angiopoietin system and determine if increasing COVID-19 disease severity leads to procoagulant changes in the endothelium. We found that plasma from patients with COVID-19 promoted a prothrombotic state in endothelial cells and that pharmacologic activation of Tie2 signaling rescued endothelial antithrombotic function. We established the presence of prothrombotic endothelial markers in lung tissue biopsies from patients with COVID-19. We next validated the relationship between COVID-19 severity and endothelial dysfunction by measuring Angpt-2 and other markers of thromboinflammatory endothelial activation in plasma from patients with mild, moderate and severe COVID-19. Markers of endothelial dysfunction and thrombosis were correlated with COVID-19 disease severity and survival. Our findings indicate a role for the Angpt-Tie2 system in endothelial dysfunction in COVID-19 pathogenesis and offer potential targets for therapeutic intervention.

## Results

### COVID-19 patient cohort.

Plasma was collected from 98 patients with PCR-confirmed SARS-CoV-2 infection: 42 were admitted to an intensive care unit (ICU), 37 were hospitalized in a non-ICU medical floor, and 19 were outpatients. In addition, we studied 12 historically healthy controls. Patient characteristics are shown in Table 1. The median time from symptom onset to sample collection was 12 days (interquartile range 7–20 days). Among hospitalized patients, the median time from hospitalization to sample collection was 5 days (interquartile range 2–8 days, Supplemental Figure 1; supplemental material available online with this article; https://doi.org/10.1172/jci.insight.151527DS1). For patients with severe COVID-19, all samples were obtained after the patient was admitted to the ICU. Patients with COVID-19 requiring ICU admission, compared with hospitalized patients not requiring ICU-level care, were more often male, but otherwise similar in terms of demographics and clinical history. Histories of obesity, cardiovascular disease, or diabetes were not associated with COVID-19 illness severity among hospitalized patients.

Anticoagulation was administered to 97.4% of hospitalized patients, with 30.3% of these patients receiving therapeutic anticoagulation at some point during hospitalization. Hydroxychloroquine was administered to 57.6% of patients, and 24.1% of patients received steroids during their admission. A total of 7 patients (8.8%) were enrolled in a trial of remdesivir. No patient received tissue plasminogen activator or tocilizumab. The majority of ICU patients (38 patients) received invasive mechanical ventilation (90.5%) and required a much longer duration of hospitalization than non-ICU patients (27.5 versus 6 median days, *P* < 0.0001). A total of 12 (28.5%) ICU patients died during their index hospitalization. All non-ICU patients (*n* = 37) survived until hospital discharge, as we excluded non-ICU patients who died during index admission or were discharged on hospice care.

### Endothelial cells treated with plasma from patients with COVID-19 exhibit upregulation of thromboinflammatory genes and promotion of coagulation on the endothelial surface, which is rescued by Tie2 activation.

**Figure 1:**
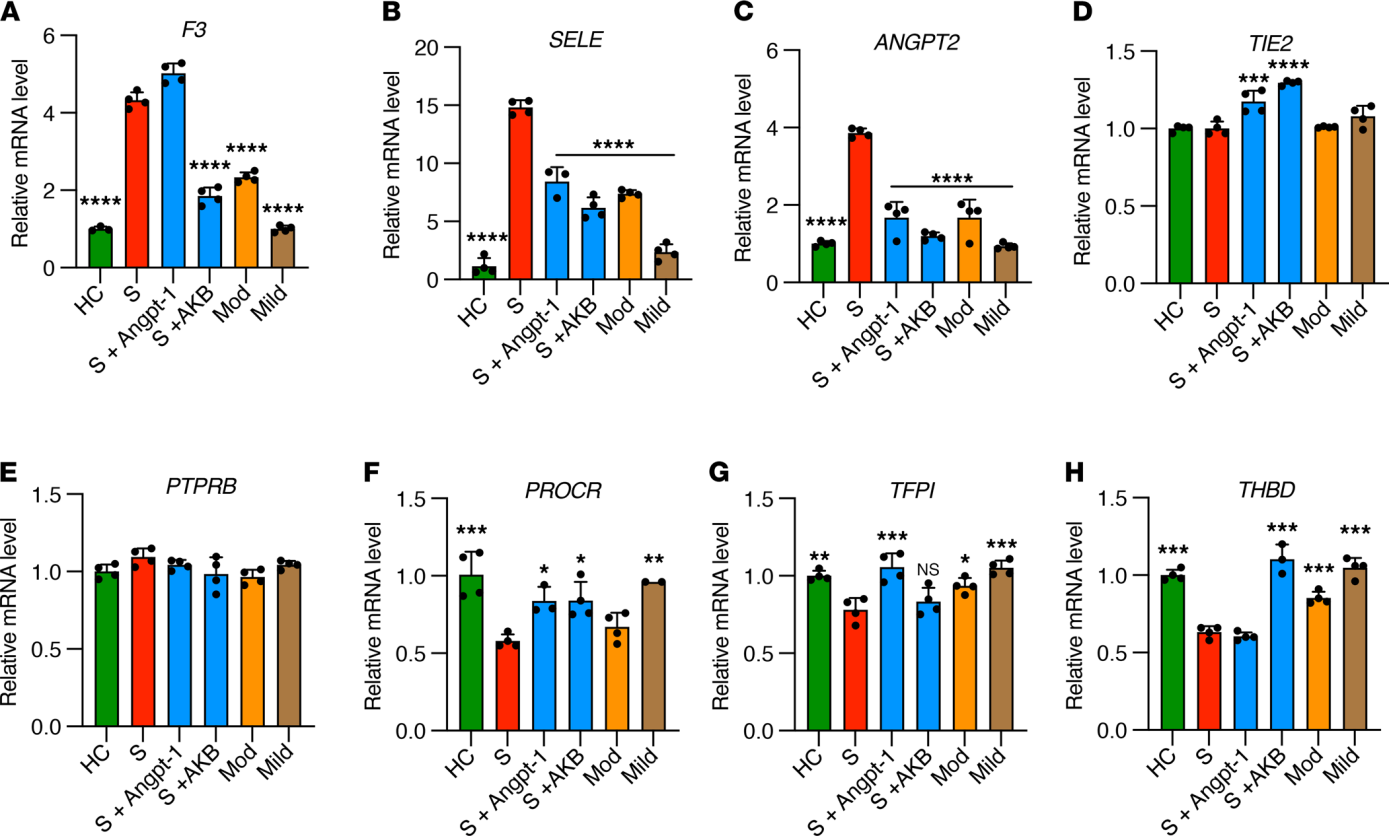
Plasma from patients with COVID-19 induces thromboinflammatory gene expression in endothelial cells. HUVECs were cultured overnight in the presence of 10% pooled plasma from patients with severe (S, ICU patients), moderate (Mod, non-ICU hospitalized patients), or mild (nonhospitalized outpatients) COVID-19 or healthy controls (HC) and analyzed for relative fold mRNA expression change of tissue factor: (**A**) *F3*, (**B**) E selectin (*SELE*), (**C**) *ANGPT2*, (**D**) *TIE2*, (**E**) VE-PTP (*PTPRB*), (**F**) EPCR (*PROCR*), (**G**) *TFPI*, and (**H**) thrombomodulin (*THBD*). Where indicated, cells were pretreated with Angpt-1 (300 ng/mL) or AKB-9778 (5 μM) for 30 minutes prior to incubation with plasma (*n* = 3–4 individual biologic replicates performed in technical duplicate). Gene expression was normalized to that of actin and changes are shown relative to HC. Graphs represent the mean ± SD. Significance in comparison with severe (S) was determined by 1-way ANOVA using Dunnett’s post hoc test, *P < 0.05, **P < 0.01, ***P < 0.001, ****P < 0.0001.

Plasma from patients with severe COVID-19 contains a wide array of proinflammatory factors including cytokines (1, 9, 10, 31), autoantibodies (11, 12), cell-free DNA (32), and NETs (13). To determine if the plasma milieu is capable of inducing procoagulant changes in the endothelium, we cultured HUVECs in the presence of 10% plasma pooled from patients with severe (ICU), moderate (non-ICU), or mild (outpatient) COVID-19 or from healthy controls. Plasma from patients with severe or moderate COVID-19 induced significant upregulation of genes encoding tissue factor, E selectin, and Angpt-2 and concurrently decreased the expression of antithrombotic genes encoding endothelial protein C receptor (EPCR), *TFPI*, and thrombomodulin (Figure 1). Plasma from patients with severe COVID-19 also induced the expression of genes regulating complement and endothelial adhesion markers (Supplemental Figure 2). Plasma from patients with severe COVID-19 did not significantly alter *TIE2* or *PTPRB* (the gene encoding VE-PTP) expression (Figure 1). Given the significant induction of Angpt-2 and its association with the coagulopathy of critical illness via inhibition of Tie2 (23), we tested whether pharmacologic activation of Tie2 could reverse thromboinflammatory gene expression triggered by COVID-19 plasma. Indeed, stimulation of Tie2 by recombinant Angpt-1 or the small molecule VE-PTP inhibitor AKB-9778 normalized many of the gene expression changes induced by plasma from patients with COVID-19 (Figure 1 and Supplemental Figure 2).

**Figure 2:**
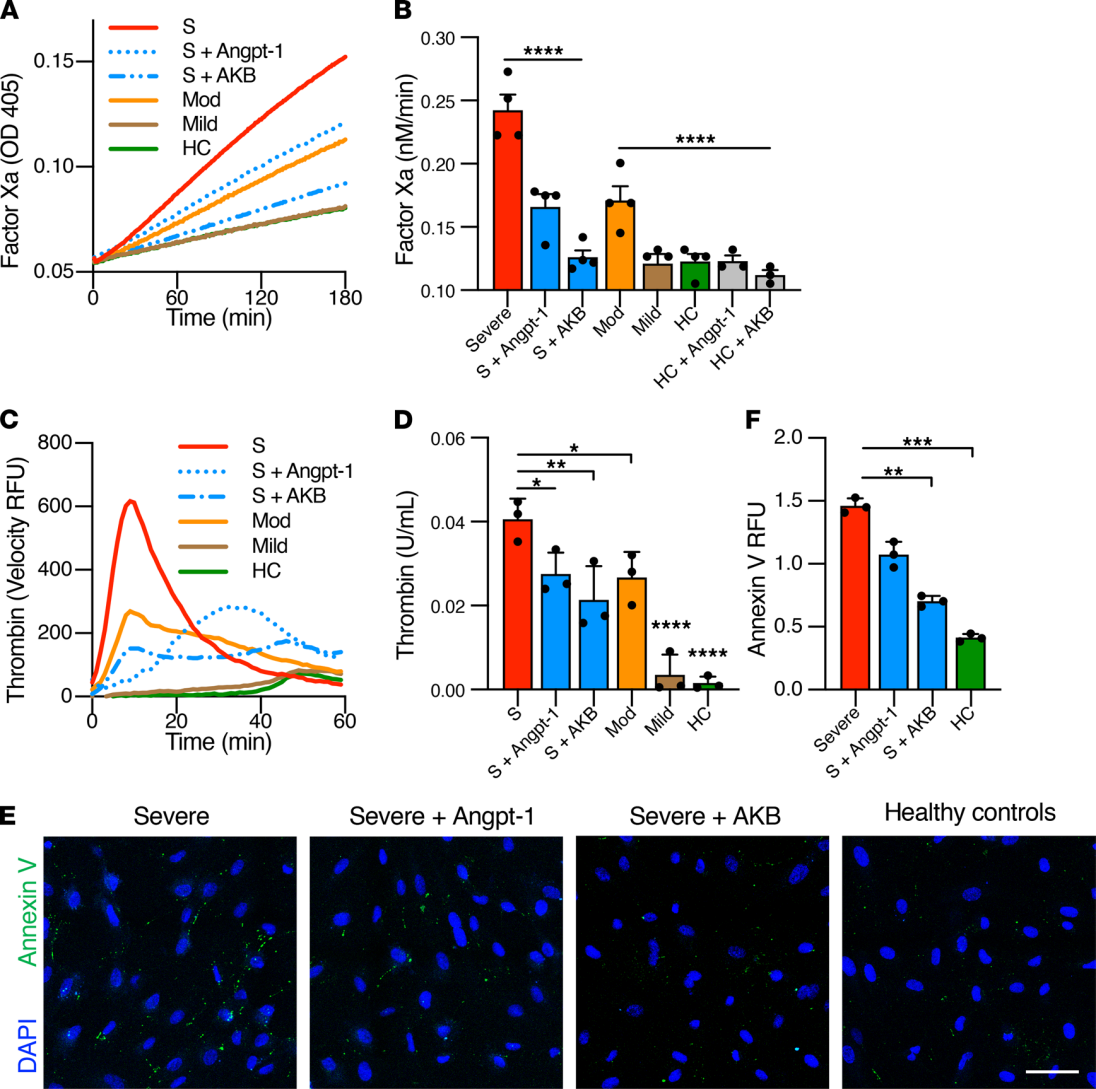
Plasma from patients with COVID-19 promotes activation of coagulation on endothelial cells. HUVECs were cultured overnight in the presence of 10% pooled plasma from patients with severe (S, ICU), moderate (Mod, non-ICU), or mild (outpatient) COVID-19 or healthy controls (HC). (**A**–**D**) Cells were analyzed for their ability to generate factor Xa or thrombin. Where indicated, cells were pretreated with Angpt-1 (300 ng/mL) or AKB-9778 (5 μM) for 30 minutes prior to incubation with plasma. (**A **and **C**) Representative experiments are depicted as mean absorbance (405 nm) or the first derivative of arbitrary fluorescence units as a function of time. (**B **and** D**) The rate of reaction for factor Xa and thrombin were converted to nM/min and U/mL, respectively, by comparison to standard curve. For factor Xa and thrombin generation assays, each data point represents the mean of 3 technical replicates, with 3–5 biologic replicates performed in total. (**E **and** F**) Cells were stained with annexin V to assess for phosphatidylserine externalization (*n* = 3 per group). Total fluorescent area was quantified and normalized for number of nuclei. (**F**) Graph represents the mean total fluorescence per 3 × 3 tile scan image ± SD. Scale bar: 50 μm. Significance was determined by 1-way ANOVA using Dunnett’s post hoc test, *P < 0.05, **P < 0.01, ***P < 0.001, ****P < 0.0001.

We further evaluated the ability of humoral factors present in plasma from patients with COVID-19 to promote procoagulant changes in the endothelium by measuring factor Xa and thrombin generation on endothelial cells following exposure to COVID-19 plasma. Factor Xa generation in this assay is dependent on expression of tissue factor, activation of tissue factor activity, and regulators of tissue factor such as TFPI. Thrombin generation is dependent on tissue factor and other regulators of procoagulant activity on endothelial cells. Tenase and prothrombinase formation are the final steps immediately preceding fibrin generation and are promoted by inflamed, but not quiescent, endothelial cells. Plasma from patients with both severe and moderate COVID-19 stimulated factor Xa generation and thrombin generation on the endothelial surface (Figure 2). The proinflammatory plasma milieu of COVID-19 may be similar to that of severe sepsis. Plasma from patients with non–COVID-19 severe sepsis and acute respiratory distress syndrome (ARDS) also promoted endothelial cell procoagulant activation in a manner similar to COVID-19 plasma (Supplemental Figure 3). Treatment with Tie2 activator Angpt-1 or AKB-9778 significantly attenuated the ability of plasma from severe COVID-19 or non–COVID-19 severe sepsis to promote procoagulant activity on endothelial cells (Figure 2 and Supplemental Figure 3). Furthermore, plasma from patients with severe COVID-19 induced externalization of phosphatidylserine, an anionic phospholipid critical for assembly of coagulation enzyme complexes (Figure 2). Treatment with Angpt-1 or AKB-9778 also reduced PS externalization in response to severe COVID-19 plasma. These results suggest that plasma from patients with moderate and severe COVID-19 promotes a prothrombotic state in endothelial cells, and that activation of Tie2 can inhibit this response.

### Increased protein markers of thrombosis in COVID-19 lung biopsies.

**Figure 3:**
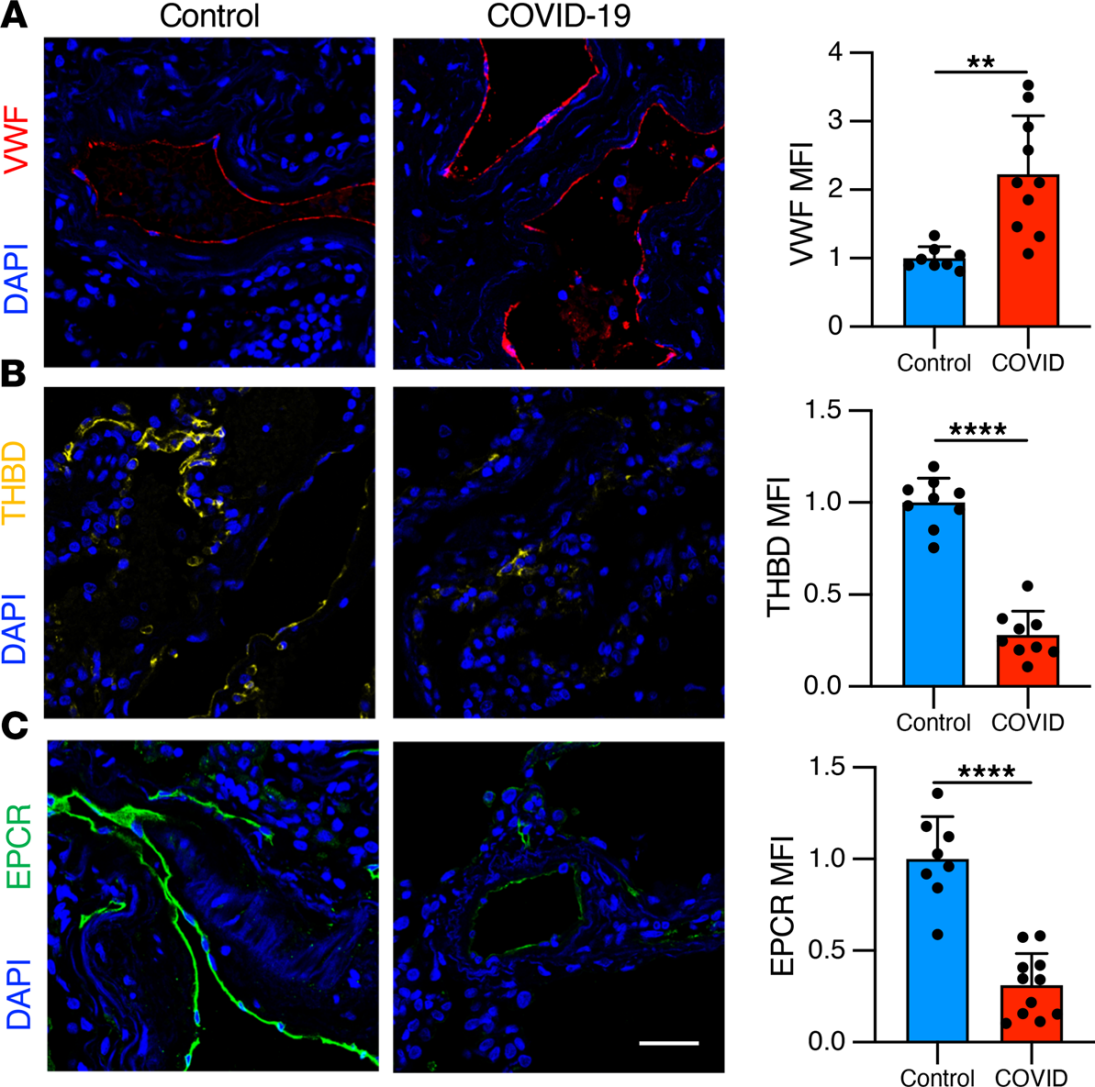
Procoagulant tissue signature of COVID-19 lung endothelium. Lung specimens were obtained during limited autopsy immediately after death in patients who died from COVID-19 (*n* = 5). Control samples (*n* = 4) were obtained from tumor-free margins of lung tumor resections and processed in identical fashion regarding timing and method of fixation. Lung specimens from patients with COVID-19 demonstrated (**A**) an increase in the prothrombotic endothelial protein vWF and (**B **and** C**) a loss of the antithrombotic factors thrombomodulin (THBD) and EPCR. In each tissue section, 2 images were obtained and mean fluorescence intensity was analyzed for each tile-scanned image and normalized to background intensity. Scale bar: 100 μm. For graphs, mean is represented by the bar with each dot as a replicate; error bars indicate SD. Significance was determined by a 2-tailed Mann-Whitney test, **P < 0.01, ****P < 0.0001.

We next sought to examine changes in expression in COVID-19 lung specimens of endothelial proteins associated with thrombotic risk. Core needle biopsies were obtained during limited autopsies performed shortly after expiration in 5 patients with COVID-19 (Supplemental Data 1 and Supplemental Table 1). Histopathologic evaluation revealed lesions similar to those described in published autopsy series — fibrin plugs within alveolar spaces and associated microvascular thrombi — suggesting that core needle sampling was representative of typical findings of COVID-19 (33). We compared these specimens to contemporaneously obtained normal lung tissue regions from tumor resections in patients without COVID-19. Despite limited COVID-19 autopsy specimen availability, we observed increased levels of the prothrombotic endothelial protein vWF and decreased levels of antithrombotic endothelial proteins EPCR and thrombomodulin (Figure 3).

### Markers of endothelial dysfunction and thrombosis are strongly correlated with COVID-19 disease severity and survival.

**Figure 4:**
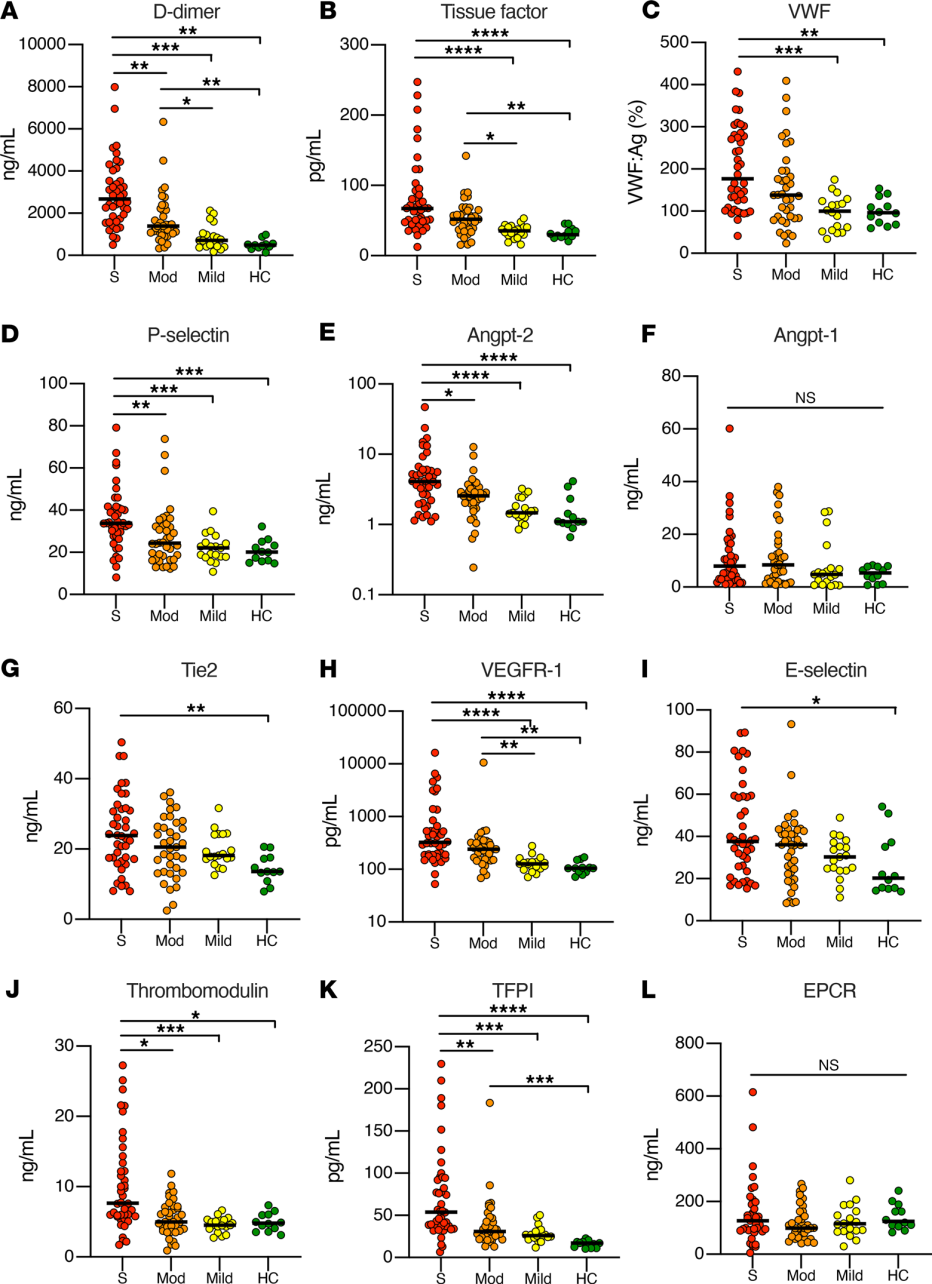
Measurement of thrombotic and endothelial markers in patients with COVID-19 by disease severity. Markers included (**A**) D-dimer, (**B**) tissue factor, (**C**) vWF, (**D**) P selectin, (**E**) Angpt-2, (**F**) Angpt-1, (**G**) Tie2, (**H**) VEGFR-1, (**I**) E selectin, (**J**) thrombomodulin, (**K**) TFPI, and (**L**) EPCR. Each data point represents individual patient plasma measurements corresponding to disease severity. Bar indicates median value. Significance among groups determined by Kruskal-Wallis with Dunn’s multiple comparison test, *P < 0.05, **P < 0.01, ***P < 0.001, ****P < 0.0001. S, severe (ICU patients); Mod, moderate (hospitalized, non-ICU); Mild (COVID-19–positive outpatients); and HC, healthy controls.

We measured the plasma concentration of proteins associated with thrombosis and procoagulant endothelial activation and correlated these values with the degree of COVID-19 severity (Figure 4). Our thromboinflammatory panel included markers of general thrombotic activation, namely D-dimer and tissue factor; markers of procoagulant endothelial activation, vWF and P selectin; and endothelial inflammation markers, E selectin and soluble vascular endothelial growth factor receptor 1 (VEGFR-1). We also investigated levels of the anti-thrombotic endothelial proteins, EPCR, TFPI, and thrombomodulin, which are cleaved from inflamed endothelium. Finally, given its role linking vascular inflammation and thrombosis, we investigated components of the Tie2-angiopoietin pathway, specifically Tie2-activating cytoprotective Angpt-1, Tie2-inhibiting proinflammatory Angpt-2, and soluble Tie2, which are also cleaved from endothelial cells during inflammatory states (25).

Several thromboinflammatory proteins were significantly higher in severe versus mild disease: tissue factor (*P* < 0.0001), vWF (*P* < 0.001), P selectin (*P* < 0.001), Angpt-2 (*P* < 0.0001), VEGFR-1 (*P* < 0.0001), thrombomodulin (*P* < 0.001), and TFPI (*P* < 0.001). In ICU compared with non-ICU patients, Angpt-2 (*P* < 0.05), P selectin (*P* < 0.01), thrombomodulin (*P* < 0.05), and TFPI (*P* < 0.01) were also significantly elevated. There were no significant differences between groups in circulating Angpt-1 and EPCR concentrations. E selectin and Tie2 were only significantly different between patients with severe COVID-19 versus healthy controls. We did not observe any overall variability in the levels of biomarkers measured in our study based on the day of hospitalization they were collected (Supplemental Figure 1).

There was significant correlation among markers of prothrombotic endothelial activation. Angpt-2 levels were highly correlated with D-dimer, E selectin, thrombomodulin, P selectin, and vWF (Supplemental Figure 4). D-dimer correlated with P selectin, tissue factor, thrombomodulin, vWF, and TFPI. The levels of many thromboinflammatory proteins were elevated in patients who developed acute kidney injury or died during their index hospitalization (Supplemental Figure 5). The relatively small number of thrombotic events, with the majority being catheter-related thrombosis, did not make it possible for us to assess the association of endothelial dysfunction and clotting events in this cohort.

**Figure 5:**
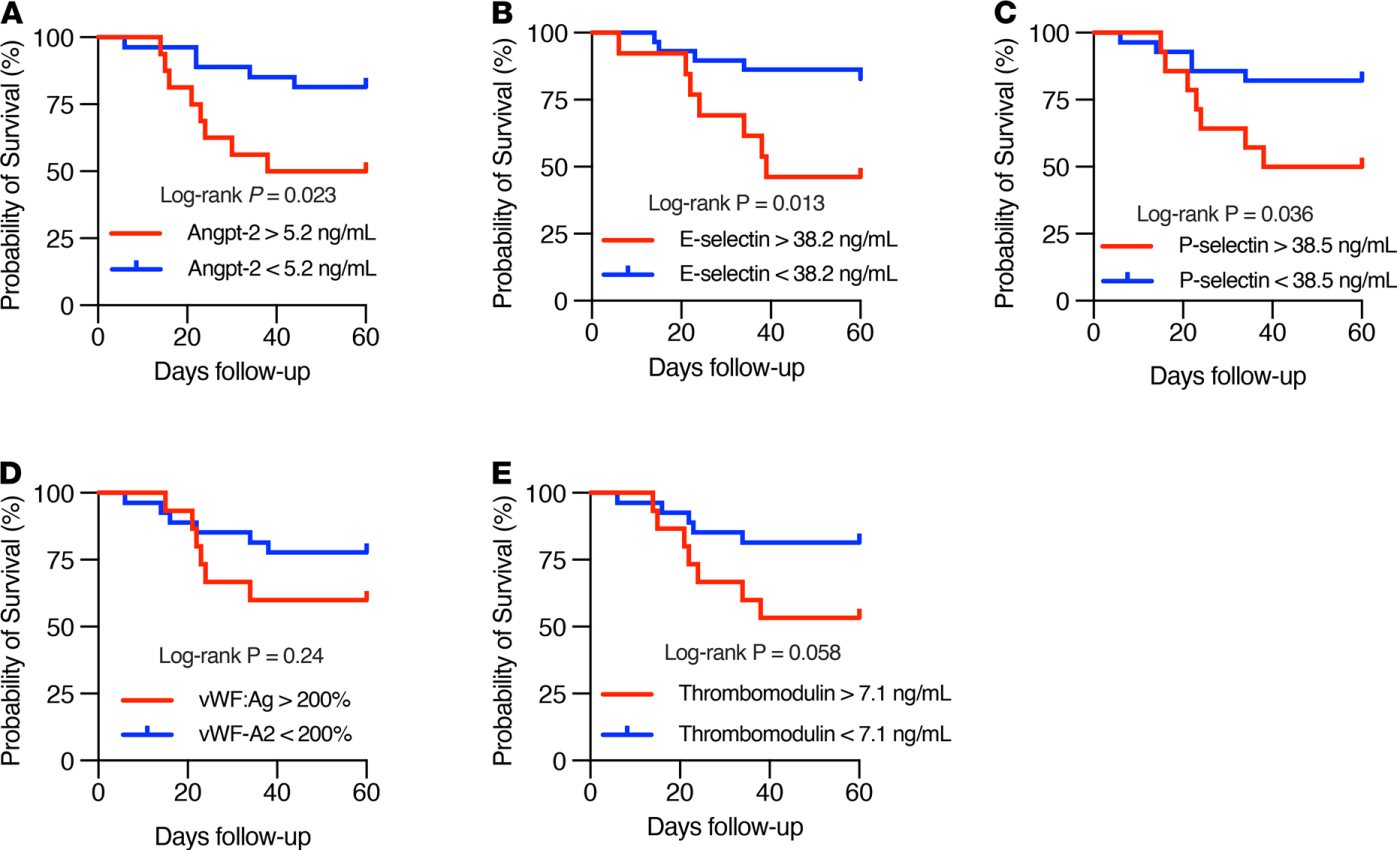
Markers of thrombotic and endothelial activation are associated with worse survival among patients with COVID-19 in the ICU. Kaplan-Meier curves of survival according to the top tertile (*n* = 14) versus bottom 2 tertiles (*n* = 28) of the plasma concentration of indicated analytes: (**A**) Angpt-2, (**B**) E selectin, (**C**) P selectin, (**D**) vWF:Ag, and (**E**) thrombomodulin. Significance was determined by log-rank test.

We performed survival analysis for selected analytes, including those that were significantly higher among patients who died during index hospitalization. Given there were 12 deaths among 42 ICU patients, we stratified ICU patients by tertiles and analyzed survival in the top tertile of analyte concentration versus the bottom 2 tertiles. Patients in the highest tertile of Angpt-2 (Log-rank *P* = 0.023), E selectin (Log-rank *P* = 0.013), and P selectin (Log-rank *P* = 0.036) had worse survival compared with patients in the bottom 2 tertiles (Figure 5). These results suggest that Angpt-2 and other markers of endothelial dysfunction and thrombosis are strongly correlated with COVID-19 disease severity and implicate perturbation of the Tie2-angiopoietin pathway in this process.

## Discussion

To interrogate the Ang2-Tie2 system in COVID-19–mediated endothelial dysfunction, we used an in vitro model in which primary endothelial cells were treated with COVID-19 plasma.

The effects of COVID-19 patient plasma on endothelial cells are notable in 2 related but distinct ways. First, there is a coordinated response such that proinflammatory and prothrombotic genes are upregulated whereas anti-inflammatory or anti-thrombotic genes are downregulated (12, 34). Second, the effects of the COVID humoral milieu extend to alterations of the endothelial cell surface that catalyze clot formation. Severe COVID-19 is associated with elevated levels of inflammatory cytokines, including TNF-α, IL-6, and IL-8 (1, 9, 10, 31), that scale with the degree of disease severity. These cytokines are known to promote expression of thromboinflammatory proteins such as tissue factor, endothelial adhesion markers, and complement (35, 36). Similar cytokine elevations are associated with other critical illnesses, including severe septic shock and cytokine release syndrome (9, 37), conditions also associated with profound endothelial injury and microvascular thrombosis. Loss of the constitutive endothelial anticoagulants such as TFPI, EPCR, and thrombomodulin is an important component of the prothrombotic endothelial transformation and may be an underappreciated driver of COVID-19 coagulopathy.

A primary function of the endothelium is to provide barrier defense to prevent excessive vessel permeability and an antithrombotic surface to promote blood circulation. Tie2 has a critical role in this process and remains activated throughout healthy adult vasculature via continuous secretion of Angpt1 from perivascular cells and platelets (24). As a receptor tyrosine kinase nearly exclusive to endothelial cells, Tie2 signaling promotes vascular quiescence by inhibiting inflammatory NF-κB (38), thus promoting expression of anticoagulant genes (39) and repressing tissue factor expression (23, 40). In our study, 2 unrelated approaches to Tie2 activation, direct Tie2 agonism with Angpt-1 and VE-PTP antagonism with AKB-9778, inhibit thromboinflammatory changes in endothelial cells induced by plasma from patients with COVID-19. This includes inhibition of genes encoding Angpt-2, endothelial adhesion markers, tissue factor, and complement C3 and factor B — all, notably, NF-κB target genes (36). This result is consistent with the ability of Tie2 activation to suppress proinflammatory endothelial phenotypes in non-COVID infectious diseases such as Gram-negative sepsis, anthrax, and malaria (23, 41, 42). Our findings support previous work suggesting that preservation of Tie2 signaling is both necessary to prevent hypercoagulation and sufficient to normalize pathological thrombosis during systemic inflammation (23). Is severe COVID-19 a Tie2-deficient state? Similar to COVID-19, severe sepsis is associated with elevated levels of both soluble Tie2 and Angpt-2, the endogenous Tie2 antagonist (23, 26, 43). Together, these findings suggest that Tie2 signaling is reduced in infectious disease states characterized by marked inflammation. However, soluble Tie2 and circulating Angpt-2 are at best an indirect assessment of Tie2 activation, and further studies are required to determine whether COVID-19 results in reduced Tie2 activation in endothelial cells. In animal models of sepsis, loss of Tie2 — both cell surface receptor and Tie2 phosphorylation — is a common feature of several infectious diseases associated with vascular leak (41). Tie2 is also expressed on a subpopulation of monocytes and recruitment and/or these cells via Angpt-2 may promote pathologic lung injury through secretion of matrix-degrading enzymes and/or inflammatory cytokines (44, 45). The role of Tie2 on nonendothelial cells is incompletely defined, and it is difficult to speculate on their role in COVID-19.

VE-PTP interacts closely with Tie2 and is a natural brake on Tie2 activity. VE-PTP is highly expressed in the lung, the organ that also bears the highest concentration of Tie2. Like the other endogenous Tie2 antagonist, Angpt-2, VE-PTP is induced by pulmonary vascular stressors such as hypoxia (27, 28). AKB-9778 (Razuprotafib) has demonstrated beneficial activity in animal models of vascular leak such as LPS-induced acute lung injury (30). Applied to cultured endothelial cells, AKB-9778 achieves ligand-independent Tie2 activation and activates Tie2 even when Angpt-1 is unable to do so during the high VE-PTP state of endothelial hypoxia. In the present study, we demonstrate that AKB-9778 strongly suppresses the procoagulant response in endothelial cells induced by COVID-19 plasma. AKB-9778 was more efficacious than Angpt-1 (Figure 2), which may reflect the ability of VE-PTP to significantly inhibit tissue factor expression or promote the weak agonist properties of Angpt-2 (46). Tie2 activation by AKB-9778 may, therefore, be an extremely effective means of dampening the thromboinflammatory state of the endothelium in COVID-19. We were surprised that Angpt-1 treatment did not reduce tissue factor (*F3*) mRNA (Figure 1), as we had previously demonstrated that Angpt-1 treatment reduced tissue factor protein in endothelial cells stimulated with LPS (23). This discrepancy may reflect differences in assay construction or inhibition of tissue factor protein, but not gene expression, by Angpt-1. TFPI is a potent negative regulator of tissue factor activity and the fact that Angpt-1 increased *TFPI* expression suggests that the inhibition of factor Xa activity in response to Angpt-1 treatment may be explained in part by increased TFPI. In contrast, AKB-9778 did not increase *TFPI* expression but did reduce *F3* (Figure 1). While both activating Tie2 signaling, Angpt-1 and VE-PTP inhibition by AKB-9778 may have different, nonredundant effects on cell signaling. In aggregate, our findings support clinical trials of AKB-9778 to improve pulmonary outcomes and mortality in moderate to severe COVID-19 (RESCUE, https://clinicaltrials.gov/ct2/show/NCT04511650). In addition to promoting endothelial barrier function to reduce the incidence or severity of acute respiratory disease syndrome, by lowering the procoagulant potential of the endothelium, AKB-9778 may also inhibit the microvascular thrombosis that is a hallmark of COVID-19 pathophysiology.

While several studies have measured circulating proteins in patients with COVID-19, few to date have examined endothelial markers in lung histopathological specimens of SARS-CoV-2 infection. We attempted to examine the expression of prothrombotic and antithrombotic endothelial proteins in situ in lung tissue from COVID-19 autopsies. Our results demonstrate upregulation of vWF and loss of thrombomodulin and EPCR in lung tissue from COVID-19. It is therefore likely that microvascular thromboses are driven at least in part by prothrombotic endothelial cell changes at the local tissue level. The profound downregulation of EPCR and thrombomodulin suggest that loss of constitutive antithrombotic function may promote clotting in COVID-19. Increased circulating thrombomodulin levels in severe COVID-19 likely reflect its cleavage from the cell surface (22). Loss of EPCR in lung specimens was unexpected because circulating EPCR was not significantly different between groups in our cohort, even comparing critical COVID-19 disease to healthy controls (Figure 5). Nevertheless, the dramatic lack of EPCR antigen in COVID-19 lung specimens suggests mechanisms other than cleavage from the cell surface, such as downregulation of gene expression, may be responsible for loss of anticoagulant endothelial proteins. Indeed, plasma from patients with severe COVID-19 inhibited expression of EPCR by nearly 50% (Figure 1).

Our plasma vascular survey demonstrates that increasing severity of COVID-19 is associated with coagulopathy and shows a clear relationship between increasing levels of endothelial cell dysfunction and increasing clinical strata of disease severity (15, 20, 21, 47–49). This study builds on prior work by demonstrating that the degree of endothelial dysfunction scales with COVID-19 severity and with procoagulant biomarkers. Specifically, markers supporting a procoagulant endothelial phenotype including Angpt-2, P selectin, thrombomodulin, and TFPI were all elevated in severe disease compared with moderate COVID-19 in our cohort. This thromboinflammatory activation of the endothelium is not unique to COVID-19. Prior data published from our group and others have demonstrated that markers of endothelial activation/dysfunction, such as Angpt-2 (23, 50), VEGFR-1 (50), E selectin (50), P selectin (51), thrombomodulin (52), vWF (53), and D-dimer (52), are elevated in sepsis, scale with disease severity, and in some cases may be predictive of survival. Previous studies suggest that circulating Angpt-2, thrombomodulin, and vWF levels are associated with adverse outcomes in COVID-19 (15, 21, 54). We expand this list to include E selectin and P selectin as well. Correlation of Angpt-2, P selectin, and vWF levels (Supplemental Figure 4) in our work and that of others suggests that Weibel-Palade body extrusion may be a common manifestation of COVID-19 endothelial cell injury. Overall, our data are in agreement with several lines of evidence suggesting that endothelial dysfunction promotes the coagulopathy of critical illness (23) and contributes to the pathobiology of severe COVID-19 (8).

Altogether, these findings suggest that a coordinated phenotypic shift of the endothelium contributes to adverse outcomes in COVID-19. Our current data add to mounting evidence that Angpt-2 is a highly sensitive and specific indicator of endothelial damage in COVID-19. Extensive research has documented that Angpt-2 is a powerful biomarker for outcomes of acute respiratory distress syndrome and sepsis (26, 55), and our current findings extend prior results implicating Angpt-2 in the coagulopathy of critical illness (23). Indeed, data suggest that Angpt-2 levels are increased in severe COVID-19 (15, 17, 19) and may predict adverse outcomes such as ICU admission (18), acute kidney injury (56), and survival (54).

Our study has several limitations. The retrospective and nonconsecutive nature of patient recruitment into the biorepositories may have inadvertently introduced bias. It would have been useful to determine whether gene expression and coagulation assays of individual plasmas correlated with biomarker measurements or could be associated with clinical outcomes. However, given the limited amount of plasma supply for the endothelial cell–based assays, it was necessary to pool plasma based on disease severity. The number of autopsy specimens was limited, which impaired our ability to perform comprehensive histopathological assessment of multiple endothelial markers, including Tie2 and phospho-Tie2. We cannot rule out that differences in specimen preparation affected antibody staining. That samples were not collected uniformly early upon hospital or ICU admission limits the applicability of our conclusions regarding endothelial biomarkers and clinical endpoints. Since samples were only collected at a single time point, we do not have data regarding how these biomarkers vary over the course of hospitalization. Limited data suggest that Angpt-2 levels increase early in hospital presentation in nonsurvivors of sepsis (26) and perhaps COVID-19 (54). Future studies may further illuminate how the levels of these biomarkers vary over time in patients with COVID-19.

In conclusion, the present study supports the concept that moderate and severe COVID-19 are driven at least in part by procoagulant endothelial cell dysfunction, the degree of which increases in parallel with COVID-19 disease severity. Elevated Angpt-2 levels may potentiate endothelial cell dysfunction through inhibition of antithrombotic Tie2 signaling. Activation of Tie2 through 2 independent mechanisms corrects the prothrombotic changes in endothelial cells induced by plasma from patients with COVID-19. Our findings support further investigation into the role of Tie2-angiopoietin in SARS-CoV-2 infection and encourage clinical trials to evaluate the efficacy of Tie2-activating therapy for treatment of more severe forms of COVID-19.

## Methods

### Patient cohort and sample collection.

Subjects in the COVID-19 cohort were recruited between April and June 2020, during the height of the COVID-19 surge, at Beth Israel Deaconess Hospital (BIDMC) by the BIDMC COVID-19 Data and Tissue Repository and at Massachusetts General Hospital (MGH) by the Massachusetts Consortium on Pathogen Readiness. Patients were recruited who had PCR-confirmed SARS-CoV-2 either in the hospital or ambulatory setting. All enrolled patients provided either written and/or verbal informed consent, participation was entirely voluntary, and the repository studies were approved by the institutional review boards (IRBs) at their respective institutions (BIDMC IRB 2020P000361 and MGH IRB 2020P000804). Non-ICU patients who died during index hospitalization or were discharged on hospice or comfort care were excluded from this study. Patients in the non–COVID-19 sepsis cohort were recruited from the Emergency Department and ICU of BIDMC. Patients were eligible if they were admitted to the hospital with a suspected infection. A medical records review was performed for relevant clinical, laboratory, and outcomes data. Patients who met clinical criteria for ARDS were included, and samples were drawn within 24 hours of emergency department presentation. All patients were enrolled under a written informed consent.

For both cohorts, blood samples were collected into ethylenediamine tetraacetic acid (EDTA) tubes and spun for 15 minutes at 2600 rpm according to standard protocol. Plasma was aliquoted into 1.5 mL cryovials and stored at –80°C, which was subsequently thawed and further aliquoted for this study. For ELISAs, individual patient plasma samples were used. For endothelial cell gene expression studies and factor Xa and thrombin generation assays on endothelial cells, pooled plasma was used. For ICU patients, 33 of 42 samples were pooled. For non-ICU patients, 27 of 37 samples were pooled. For mild and healthy controls, all the of the samples were pooled (19 and 12, respectively). For pooling, we used plasma samples that had sufficient volume remaining after performing biomarker measurements. Pooled plasma was aliquoted to allow for the single use of a thawed vial for subsequent endothelial cell experiments.

Demographics, laboratory values, and clinical endpoints were assessed through reviews of the patients’ electronic medical records. Acute kidney injury was defined as a rise in serum creatinine of more than 0.3 mg/dL within 48 hours or an increase of greater than 1.5 times the baseline over 7 days, and patients with end-stage renal disease were excluded from this analysis. Thrombotic events were reviewed, including deep vein thrombosis, pulmonary embolism, catheter-related thrombosis, and clinically documented thrombosis at another location.

### Plasma biomarker measurements.

Plasma samples from individual patients were thawed on ice, then centrifuged for 5 minutes to pellet any debris. Concentrations of the following proteins were measured using a Luminex Human Premixed Multi-Analyte Kit (R&D Systems) for the following analytes: Angpt-1, Angpt-2, D-dimer, E selectin, P selectin, thrombomodulin, Tie2, tissue factor, vWF, and VEGFR-1, according to the manufacturer’s protocol. Samples were diluted 1:2 and run on a MAGPIX system (MilliporeSigma), which was preprogrammed according to kit specifications. For EPCR, plasma samples were analyzed by ELISA (Diagnostica Stago) at 1:51 dilution according to the manufacturer’s protocol. For TFPI, plasma samples were analyzed by ELISA (R&D Systems) at a 1:100 dilution according to the manufacturer’s protocol.

### Human lung tissue isolation and immunofluorescence microscopy.

Core needle biopsies were obtained during limited autopsies of patients with COVID-19 within 3 hours of patient expiration. For controls, surgical specimens from patients undergoing lung tumor resections were analyzed by a pathologist, and lung tissue within the tumor-free margins was isolated. These controls were selected due to the similar nature in which the lung tissue was processed compared with a standard autopsy where there is a longer delay before tissue is placed in fixative. Tissue was washed and fixed in freshly prepared 4% paraformaldehyde for 24 hours and transferred to 70% ethanol. Samples were embedded in paraffin within 7–10 days and cut into 6 μm thick sections for staining. Primary antibodies used for immunofluorescence microscopy are as follows: anti-vWF (clone IIIE2.34, MilliporeSigma), anti-thrombomodulin (clone 141C01, Thermo Fisher Scientific), and anti-EPCR (clone LMR-42, Thermo Fisher Scientific). Images were obtained using a Zeiss LSM 880 upright laser scanning confocal microscope in 3 × 3 tile-scan mode with a Plan-Apochromat 40X/1.3 Oil DIC M27 objective.

### Endothelial cell culture.

HUVECs (pooled donor, Lonza) were grown and maintained in endothelial cell growth basal media (EBM-2, Lonza), containing 2% FBS and contents of the EGM-2 SingleQuots growth factor supplement kit. Cells from passages 3–5 were used for experiments. HUVECs were grown to confluency in 96-well plates (Corning) and incubated overnight with 10% pooled patient plasma and H-Gly-Pro-Arg-Pro-OH (GPRP, 5 mM, Cayman Chemical) in the presence of complete growth media. Supplementation of Angpt-1 (300 ng/mL, R&D Systems) or AKB-9778 (5 μM, a gift from Aerpio Pharmaceuticals, Inc.) was performed 30 minutes prior to addition of patient plasma. All endothelial cell–based studies were performed in a dedicated tissue culture room designated specifically for work with COVID-19 biospecimens.

### Gene expression analysis.

Confluent HUVECs were treated with pooled patient plasma as described above, and gene expression was determined using a 2-step Cell-to-Ct Taqman kit (Thermo Fisher Scientific). The following gene expression probes (Taqman, Thermo Fisher Scientific) were used: Hs00174057_m1 (*SELE*), Hs00264920_s1 (*THBD*), Hs00409207_m1 (*TFPI*), Hs00197387_m1 (*PROCR*), Hs01076029_m1 (*F3*), Hs00169867_m1 (*ANGPT2*), Hs00176096_m1 (*TEK*), Hs00160781_m1 (*PTPRB*), Hs00156060_m1 (*CFB*), Hs00157263_m1 (*CFD*), Hs00163811 (*C3*), Hs01004342_m1 (*C5*), Hs00164932_m1 (*ICAM1*) Hs01003372_m1 (*VCAM1*), and Hs00194899_m1 (*ACTB*). Quantitative reverse transcriptase–PCR (qrt-PCR) was performed in technical duplicate for each biologic sample using a QuantStudio 6 Flex real-time PCR system (Applied Biosystems). Gene expression was compared with *ACTB* control and normalized to cells treated with healthy control plasma using the ΔΔCt method.

### Factor Xa and thrombin generation assays.

HUVECs treated as above were equilibrated at room temperature and treated with calcium inophore A23187 (2–6 μM, MilliporeSigma) for 15 minutes to promote tissue factor activation. Cells were washed twice with HEPES-buffered saline (HBS-BSA) consisting of 20 mM HEPES at pH 7.4 (Gibco),150 mM NaCl, 5 mM KCl, and fatty acid–free BSA (1%, MilliporeSigma). For Factor Xa generation experiments, cells were incubated with HBS-BSA containing factor X (125 nM, Haematologic Technologies, Factor VIIa (0.6 nM, Haematologic Technologies), and chromogenic substrate biophen-CS11 (22) (150 μM, Anaira Diagnostica). Absorbance at 405 nm was measured every minute for 3 hours on an xMark Spectrophotometer (Bio-Rad). Maximal reaction velocity was converted to FXa nM/min based on standard curve analysis of FXa (Haematologic Technologies) serial dilutions. For thrombin generation experiments, cells were washed twice with HBS-BSA and incubated in 80 μL HBS plus 20 μL pooled human plasma (George King Bio-Medical) to supply coagulation factors and 5 mM GPRP. Thrombin generation was measured using the fluorogenic substrate Boc-L-FPR-ANSNH-C_2_H_5_ (SN-20, Haematologic Technologies). Fluorescence (excitation 352 nm/emission 470 nm) was measured every minute for 1 hour using the Synergy HTX plate reader (BioTek). The first derivative of thrombin generation curves was compared with a standard curve of thrombin to determine thrombin generation in U/mL. All biologic replicates were performed in technical triplicate.

### Phosphatidylserine externalization.

HUVECs were grown to confluence in glass chamber slides and incubated with pooled patient plasma as described above. Cells were washed with 10 mM HBS (pH 7.4) containing 140 mM NaCl, 2.5 mM CaCl_2_, and 2% FBS (annexin V binding buffer) and stained with annexin V-Alexa Fluor 488 (Thermo Fisher Scientific) at a 1:50 dilution and Zombie Red viability dye (BioLegend) at a 1:1000 dilution for 15 minutes at room temperature in annexin V binding buffer. Cells were washed and fixed in annexin V binding buffer containing 4% paraformaldehyde for 7 minutes. Cells were washed 3 times and mounted with DAPI. Images were obtained using a Zeiss LSM 880 upright laser scanning confocal microscope in 3 × 3 tile-scan mode with a Plan-Apochromat 20×/0.8 M27 objective.

### Image analysis.

Fluorescent images were analyzed using ImageJ software (NIH). For phosphatidylserine externalization, the threshold for annexin V staining was adjusted and the total fluorescent area was normalized to the number of nuclei. For immunofluorescence microscopy of lung sections, fluorescence intensity was quantified per tissue area, after measuring and subtracting background signals from each image.

### Statistics.

Tests of normality were performed using the Anderson-Darling and D’Agostino-Pearson method. Statistical significance for binary comparisons of continuous variables were assessed by unpaired 2-tailed Student’s *t* test unless the data did not demonstrate normality, in which case differences between groups were analyzed by the Mann-Whitney *U* test. For comparison of continuous variables across multiple groups, none of which passed the test of normality, with the exception of Tie-2, the Kruskal-Wallis test with Dunn’s post hoc for multiple comparisons was performed. Correlation matrix analysis was performed using 2-tailed nonparametric Spearman correlation. Survival analysis for ICU patients was performed by segregating patients into the top tertile versus bottom 2 tertiles for each analyte. Kaplan-Meier analysis was performed, and survival was compared for the top tertile versus bottom 2 tertiles using the Mantel-Cox log-rank test. Categorical variables were compared using Fisher’s exact test. All statistical analysis was performed using GraphPad Prism (version 9.0; GraphPad Software). *P* values of less than 0.05 were considered significant.

### Study approval.

This study measuring biomarkers and associated clinical data in patients enrolled in the BIDMC COVID-19 Data and Tissue Repository was approved by the BIDMC IRB (protocol 2020P000621). For all COVID-19 autopsy studies, informed consent was received for limited autopsies by a pathologist during a witnessed phone call immediately after death of the patient. Research using autopsy tissue was approved by the BIDMC institutional review board (IRB 2020P000525). A HIPPA waiver was granted to access the medical records of the patients undergoing autopsy (IRB 2020P00412).

## Author contributions

AAS, RF, and SMP conceived the study. AAS performed endothelial cell studies, immunofluorescence studies, measured analytes in plasma from COVID-19 patients, and analyzed biologic and clinical data. SMC performed endothelial cell culture and reagent preparation. GMPH collected, organized and analyzed clinical data. ZJM, KDS, VB, and JQ collected clinical data. AYC, DN, DHB, REG, and XGY oversaw collection of biorepository specimens and clinical data from the COVID-19 cohort. KP, NIS, and AKN contributed new reagents and analytic tools. AAS, RF, and SMP wrote the manuscript with input and approval from all authors.

## Supplementary Material

Supplemental data

## Figures and Tables

**Table 1:**
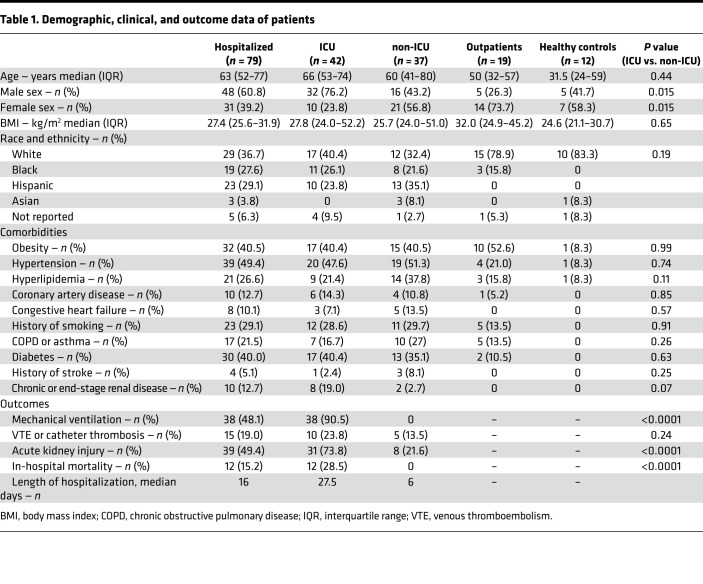
Demographic, clinical, and outcome data of patients

## Version 1 09/10/2021

In-Press Preview

## Version 2 10/22/2021

Electronic publication
